# Acute and Prolonged Adverse Effects of Temperature on Mortality from Cardiovascular Diseases

**DOI:** 10.1371/journal.pone.0082678

**Published:** 2013-12-09

**Authors:** Yu-Kai Lin, Chin-Kuo Chang, Yu-Chun Wang, Tsung-Jung Ho

**Affiliations:** 1 Environmental and Occupational Medicine and Epidemiology Program, Department of Environmental Health, Harvard School of Public Health, Boston, Massachusetts, United States of America; 2 Institute of Environmental Health, National Taiwan University College of Public Health, Taipei, Taiwan; 3 Psychological Medicine Department, King’s College London, Institute of Psychiatry, London, United Kingdom; 4 Department of Bioenvironmental Engineering, College of Engineering, Chung Yuan Christian University, Jhongli, Taiwan; 5 Research Center for Environmental Risk Management, Chung Yuan Christian University, Jhongli, Taiwan; 6 The Division of Chinese Medicine, China Medical University Beigang Hospital, Yunlin, Taiwan; 7 School of Chinese Medicine, College of Chinese Medicine, China Medical University, Taichung, Taiwan; The Ohio State University, United States of America

## Abstract

**Background:**

Cardiovascular diseases are the leading causes of death worldwide, especially for developed countries. Elevated mortality from cardiovascular diseases has been shown related to extreme temperature. We thus assessed the risk of mortality from cerebrovascular diseases, heart diseases, and ischemic heart disease (IHD) in relation to temperature profiles in four subtropical metropolitans (Taipei, Taichung, Tainan, and Kaohsiung) from 1994 to 2007 in Taiwan.

**Methods:**

Distributed lag non-linear models were applied to estimate the cumulative relative risks (RRs) with confidence intervals of cause-specific mortality associated with daily temperature from lag 0 to 20 days, and specific effect of extreme temperature episodes with PM_10_, NOx, and O_3,_ and other potential confounders controlled. Estimates for cause-specific mortalities were then pooled by random-effect meta-analysis.

**Results:**

Comparing to centered temperature at 27°C, the cumulative 4-day (lag 0 to 3) risk of mortality was significantly elevated at 31°C for cerebrovascular diseases (RR = 1.14; 95% CI: 1.00, 1.31) and heart diseases (RR =  1.22; 95% CI: 1.02, 1.46) , but not for IHD (RR =  1.09; 95% CI: 0.99, 1.21). To the other extreme, at 15°C, the cumulative 21-day (lag 0 to 20) risk of mortality were also remarkably increased for cerebrovascular diseases, heart diseases, and IHD (RRs  =  1.48 with 95% CI: 1.04, 2.12, 2.04 with 95% CI: 1.61, 2.58, and 1.62 with 95% CI: 1.30, 2.01, respectively). Mortality risks for cardiovascular diseases were generally highest on the present day (lag 0) of extreme heat. No particular finding was detected on prolonged extreme temperature event by pooling estimations for cause-specific mortality.

**Conclusions:**

Low temperature was associated with greater risk of mortality from cardiovascular diseases in comparison with that of high temperature. Adverse effects of extreme temperatures are acute at the beginning of exposure.

## Introduction

Numerous studies reported increased mortality risk associated with the variations of daily ambient temperature, in which the relationship has been characterized as U- or V- or J-shaped curves [1], [2].

Exposure to high temperature could increase plasma viscosity and cholesterol levels in serum, resulting in higher blood pressure [3]. Excess mortality from cardiovascular diseases has been found significantly associated with extremely high temperature [4], [5]. In addition, increased mortality during heat waves has been attributed mainly to cardiovascular conditions and cerebrovascular disorders [6].

Moreover, low temperature could stress cardiovascular system due to fluctuations of blood pressure and hematological changes following cold-induced vasoconstriction and consequent loss of plasma fluid, which both predispose the subject to arterial thrombosis [7]. The negative association between cardiovascular deaths and ambient temperature was reported [8], [9], however, some other conflicting outcomes were also revealed [10], [11].

Cardiovascular diseases are the leading causes of death worldwide, contributing almost 32% of all death in women and 27% in man in 2004 [12]. The occurrence of mortality from cardiovascular diseases could be acute [13], and frequently happens before the admission into hospitals [14]. Therefore, the issue of sudden deaths from cardiovascular diseases should be of public health importance for all the authorities. Further research to characterize the risk factors of mortality from cardiovascular diseases is warranted [15].

Taiwan locates in subtropical area with an annual average temperature of 24°C. The association between mortality of all cardiovascular diseases and daily ambient temperature has been reported in some studies with Taiwanese population [16]–[19]. However, except studies conducted in Europe and China [20], [21], the association between mortality from specific cardiovascular diseases and ambient temperature remained unclear to date, especially in subtropical areas. Moreover, few studies depicted a intricate exposure-response relationship or lagged effect of low temperature on cause-specific mortality from cardiovascular diseases [20]. Therefore, this study aimed to evaluate the acute and prolonged effects of extreme temperatures on mortality of cerebrovascular diseases, heart diseases, and ischemic heart disease in four major metropolitans (Taipei, Taichung, Tainan, and Kaohsiung) of Taiwan.

## Materials and Methods

### Data sources

**Figure 1:**
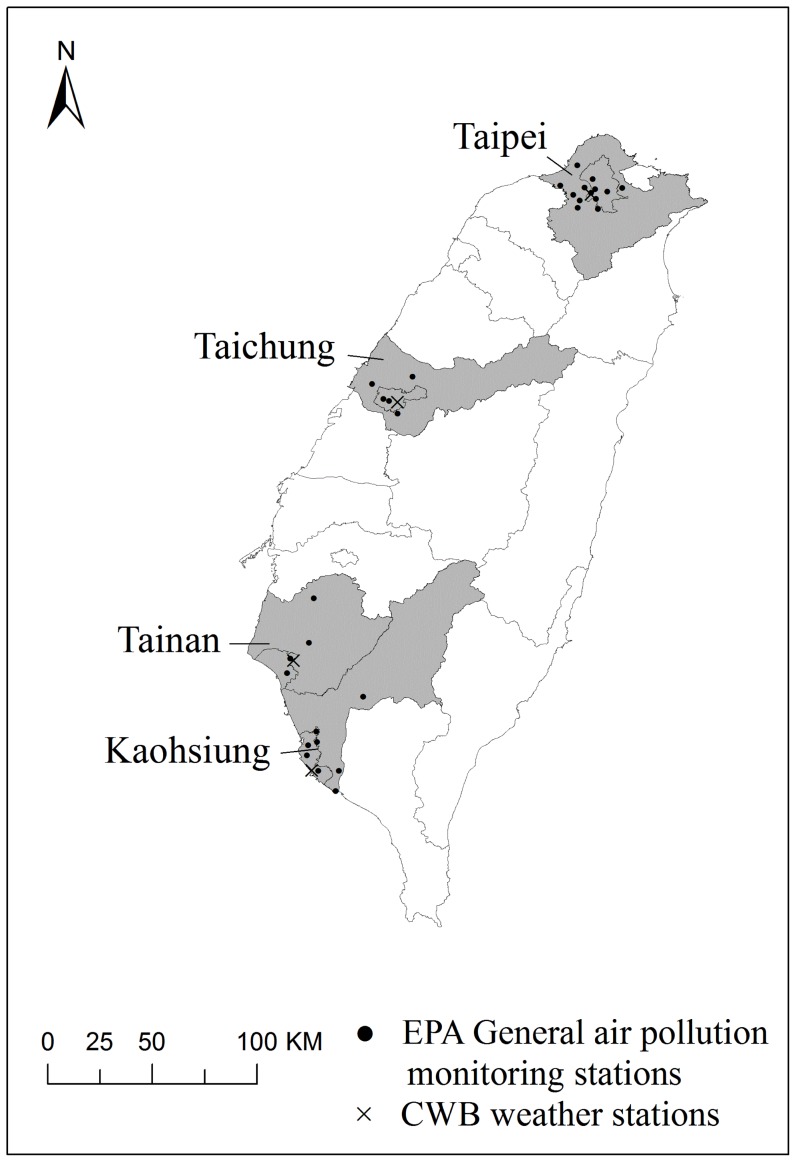
Study area and locations of general air monitoring and surface weather stations in Taiwan during 1994–2007.

The present study utilized data of vital statistics obtained from Department of Health, daily meteorological records from the Central Weather Bureau, and daily air pollution monitoring records from the Environmental Protection Administration for metropolitan Taipei, Taichung, Tainan, and Kaohsiung in Taiwan from 1994 to 2007. The metropolitans involved in our analyses, comprising 63.7% of population in the whole nation, are located respectively from north to south in Taiwan Island (Fig. 1).

Causes of death were coded according to diagnoses of the 9th revision of International Classification of Diseases (ICD-9). The records of mortality from cerebrovascular diseases (ICD-9: 430-438), heart diseases (ICD-9: 391, 402, 404, 415, 416, 785, 393-398, and 420-429), and IHD (ICD-9: 410-414) during the study period were retrieved from the death register database established by Department of Health, Taiwan.

Taiwan Central Weather Bureau provides 24-hour weather information, including average temperature, maximum temperature, minimum temperature, relative humidity, wind speed, and barometric pressure, from 25 real-time weather monitoring stations all around Taiwan [22]. Current analyses used daily weather measurements from the Taipei, Taichung, Tainan, and Kaohsiung weather stations collected from the beginning of 1994 to the end of 2007 [22].

Taiwan Air Quality Monitoring Network, established by the Taiwan Environmental Protection Administration Agency in 1993, consists of 74 stationary monitoring stations distributed throughout the island [23], [24]. Concentrations of ambient air pollutants, including particulate matters less than 10 µm in aerodynamic diameter (PM_10_), nitrogen dioxide (NO_2_), and ozone (O_3_), are measured and recorded hourly at each station. We analyzed the daily average data for PM_10_, O_3_, and NO_2_ monitored at 13, 5, 4 and 10 general ambient stations in Taipei, Taichung, Tainan, and Kaohsiung metropolitans, respectively. Figure 1 shows the locations of the weather and ambient environment stations.

### Definition of extreme temperature events

To assess the additional effects of prolonged extreme temperature (“events”), we created a variable *Extreme* which described each day of the study period as normal temperature or extreme heat or cold. The risk of cause-specific death associated with six types of extreme temperature events consisting of temperatures in the 97^th^ (n =  114 days) or 5^th^ (n =  187 days) percentile lasting for 3–5, 6–8, or >8 days was estimated. All the days not included in an event was then classified as normal.

### Non-linear association between daily temperature and cause-specific death, and the risk estimation for extreme temperature events

The association between the daily average temperature and daily cause-specific death was evaluated using a distributed lag non-linear model (DLNM) with Poisson distribution. Natural cubic spline (NS) DLNM models were used to analyze the non-linear and delayed effects of temperature and air pollutants. The cross-basis function contains the dimensions of variables and lag days. This study placed the knots of variables at equally spaced quantiles of the predictor, and the knots of lag at equally spaced values on the log scale of lags. Relative risks (RR) due to temperature and air pollutants were estimated using the cross-basis function in DLNM models, as what described above [25], [26].

The covariate “daily average temperature” was set at NS with 5 degrees of freedom (*df*). To estimate the effects of ambient temperature, the cumulative RR and 95% confidence interval (CI) of cause-specific death were estimated by comparing the risk associated with the extreme temperatures of 15°C and 31°C with that at the temperature resulting in the lowest cause-specific diseases deaths (the centered temperature). In study metropolitans, 15°C is about 1-8th percentile measurement and the 31°C is about 97-99.5th percentile measurement of average temperature that varied from Northern to Southern Taiwan.

Previous studies indicated the effects of cold may last for more than 21 days [27], [28], because of mortality displacement. However, the mortality risk of heat generally occurred immediately (peak RR at lag 0) and last less than 5 days [29]. Therefore, cumu`lative 4-day (lag 0 to lag 3, lag set at 3 *df*) RR was estimated to represent the acute effect of high temperature of 31°C, and cumulative 21-day (lag 0 to lag 20, lag set at 4 *df*) RR was estimated to represent the prolonged effect of low temperature of 15°C.

The previously defined extreme temperature events (*Extremes*) was set as categorical covariates and the risk for these events estimated by comparison with the risk on days defined as normal temperature (nonconsecutive days of extreme temperature or days of normal temperature).

The concentration of the air pollutants PM_10_, NO_2_, and O_3_ was set at NS with 5 *df.* Six-day cumulative effects (lag 0 to lag 5, 3 *df* for lag space) were estimated by comparing concentrations of air pollutants at the 75th (Q3) percentile with the concentrations at the 25th (Q1) percentile.

The model for the expected cause-specific death on day (t) for city (c) is



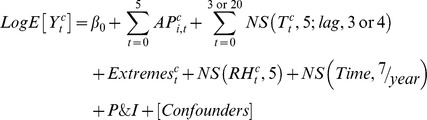
where 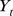 is the expected cause-specific death on day t, 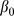 represents the intercept, and 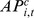 is the NS for measurements of air pollutants (*i* = 1–3 for PM_10_, NO_2_, and O_3_, *df* =  5) on day t, and effects were accumulated for 6 days (lag day 0 to lag day 5) with the lag space set at 3 *df*. 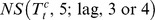 is the NS for the daily average temperature. The temperatures were set at 5 *df* and their effects accumulated for 4 days (lag day 0 to lag day 3) with the lag space set at 3 *df* or 21 days (lag day 0 to lag day 20) with the lag space set at 4 *df*. 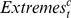 is the categorical variable representing extreme temperature events on day t. The NS function with 5 *df* was also used in the daily measurement of relative humidity (RH). The smoothing time term (*Time*) was set at 7 *df* per year. Other covariates, such as holidays, day of a week, and daily death for pneumonia and influenza (*P&I*, ICD-9: 480-487) were also adjusted in the models.

Sensitivity analysis was used to evaluate *df*, which ranged from 4 to 6 for the temperature-mortality curves. Time smoothing with *df*s of 4, 7, and 14 per year was also performed. Akaike’s information criterion was used for model selection, in which a lower value indicates a better model [30].

### Random-effect meta-analysis

City-specific RRs of cause-specific mortality associated with temperature and extreme temperature events were further evaluated for combined effects using a meta-analysis method [31]. The restricted maximum likelihood was set as an estimator of the amount of heterogeneity.

All data manipulation and statistical analyses were performed by SAS version 9.1 (SAS Institute Inc., Cary, NC, USA) and Statistical Environment R 2.15 (packages: dlnm and metafor)**.**

## Results

Table 1 lists the characteristics of the ambient environment and cause-specific death for study metropolitans. The mean temperature are about 23–25°C in these four metropolitans. Average temperature and concentrations of PM_10_ and O_3_ are higher in Southern Taiwan. The group of cerebrovascular diseases is the major leading cause of death (with the highest average numbers in all these four metropolitans) among the diseases of research interest.

**Table 1:** Characteristics of the daily ambient environment and cause-specific death in study metropolitans from 1994 to 2007.

	Taipei			Taichung			Tainan			Kaohsiung		
	Mean	Range	S.D	Mean	Range	S.D	Mean	Range	S.D	Mean	Range	S.D
Temperature (°C)	23.2	8.10—33.0	5.28	23.7	8.10—32.0	4.70	24.6	9.20—31.7	4.54	25.3	10.5—32.0	3.82
Relative Humidity (%)	76.1	47—100	9.07	74.8	41—99	7.66	76.8	48—100	6.95	76.0	46—100	7.07
PM_10_ (µg/m^3^)	49.7	12.3—222	23.8	62.5	10.9—255	30.9	70.6	12.3—299	35.8	80.1	18.3—225	39.7
NO_2_ (ppb)	36.0	4.65—145	15.7	32.4	3.15—108	14.5	23.9	1.76—73.8	10.5	32.5	6.11—86.7	14.5
O_3_ (ppb)	23.7	3.35—64.9	8.87	23.3	2.26—75.0	9.48	26.4	2.23—76.5	10.4	26.8	2.17—75.2	12.2
Cerebrovascular diseases (n.)	8.09	0—24	3.06	3.22	0—13	1.86	3.22	0—13	1.87	3.56	0—13	1.94
Heart Diseases (n.)	3.75	0—14	2.15	1.32	0—8	1.21	1.45	0—10	1.25	1.62	0—9	1.30
Ischemic heart disease (n.)	4.40	0—15	2.27	1.25	0—7	1.13	1.45	0—9	1.24	1.84	0—9	1.39
Pneumonia and Influenza (n.)	2.39	0—13	1.74	0.98	0—8	1.05	1.29	0—9	1.21	1.55	0—10	1.36

Note: S.D: standard deviation; PM_10_: particles less than 10 micrometers in diameter; NO_2_: nitrogen dioxides; O_3_: ozone.

### Cumulative 4-day and 21-day temperature effects

The effects of average temperatures and extreme temperature events on cause-specific mortality were estimated using DLNM after controlling for daily average levels of PM_10_, NO_2_, O_3_, RH, daily death for pneumonia and influenza, holidays, day of a week, and long-term trends. The lowest mortality from cardiovascular diseases was associated with an average temperature of 27°C (i.e. the baseline temperature that was used to estimate the risk of mortality associated with extreme temperature at 15 and 31°C) among study metropolitans.

**Figure 2:**
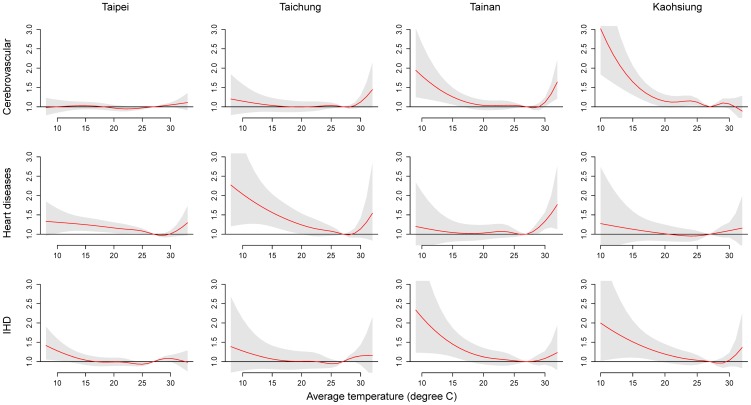
Associations between mortality from cerebrovascular diseases, heart diseases, and ischemic heart disease and daily average temperatures in metropolitans Taipei, Taichung, Tainan, and Kaohsiung during 1994–2007. Cumulative 4-day (lag 0 to 3) RRs were estimated using DLNM with a centered temperature of 27°C.

This study analyzed with maximum lag 3 for extreme heat and lag 20 for extreme cold to observe the time course of temperature effect. Figure 2 shows the cumulative 4-day RRs of cause-specific mortality in association with the daily average temperature. Acute effect of high temperature was significant on mortality from cerebrovascular diseases and heart diseases in Tainan. In addition, acute effects of low temperature were highest on mortality from heart disease in Taipei and Taichung, IHD in Tainan, and cerebrovascular diseases in Kaohsiung.

**Figure 3:**
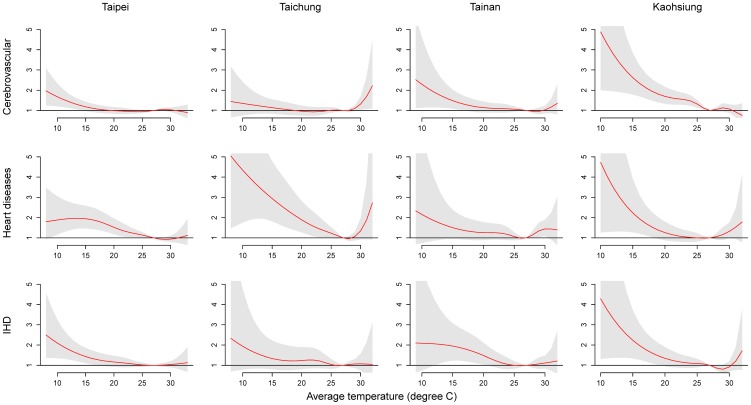
Associations between mortality from cerebrovascular diseases, heart diseases, and ischemic heart disease and daily average temperatures in metropolitans Taipei, Taichung, Tainan, and Kaohsiung during 1994–2007. Cumulative 21-day (lag 0 to 20) RRs were estimated using DLNM with a centered temperature of 27°C.

Figure 3 presents the cumulative 21-day RRs of cause-specific mortality in association with the daily average temperature. In general, low temperature was associated with greater risk of cause-specific mortality in comparison with that of high temperature; prolonged cold effects were particularly significant in Taipei, Taichung, and Kaohsiung. Prolonged effects of high temperature on mortality from cerebrovascular diseases in Taichung and heart diseases in Tainan were also significant.

### Delayed effects of temperature

**Figure 4:**
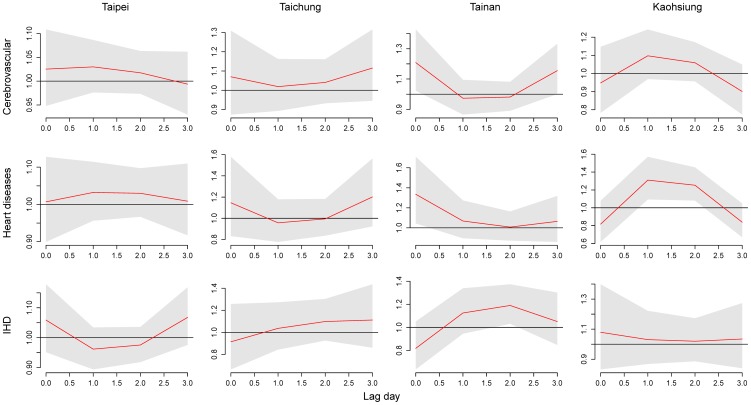
Mortality risks for cerebrovascular diseases, heart diseases, and IHD associated with an ambient temperature of 31°C compared to a centered temperature of 27°C on lag 0 to 3 in metropolitans Taipei, Taichung, Tainan, and Kaohsiung during 1994–2007.

Figure 4 shows the RRs for cause-specific mortality in different lags at 31°C. The RRs were highest at lag 0 for heart disease (RR  =  1.34, 95% CI: 1.04, 1.71) and cerebrovascular diseases (RR  =  1.21, 95% CI: 1.02, 1.43) in Tainan.

**Figure 5:**
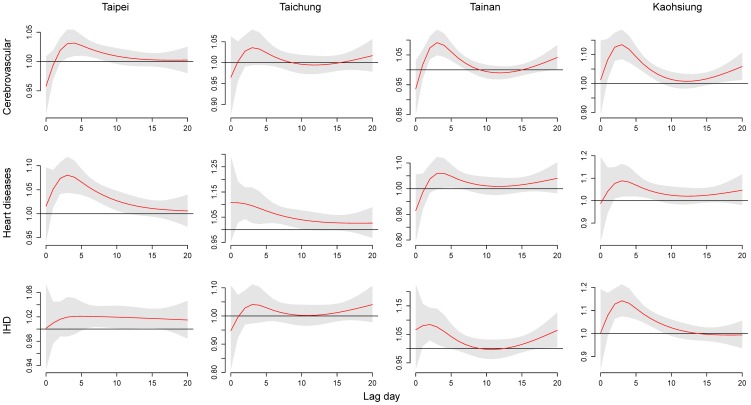
Mortality risks for cerebrovascular diseases, heart diseases, and IHD associated with an ambient temperature of 15°C compared to a centered temperature of 27°C on lag 0 to 20 in metropolitans Taipei, Taichung, Tainan, and Kaohsiung during 1994–2007.

Figure 5 presents the RRs for cause-specific mortality for various lags at 15°C. Except those for Taipei, risks generally peaked at lag 0. The RRs were highest at lag 0 for cerebrovascular diseases (RR =  1.13, 95% CI: 1.08, 1.19) and IHD (RR =  1.14, 95% CI: 1.07, 1.21) in Kaohsiung.

### Random-effect meta-analyses for temperature effect

**Figure 6:**
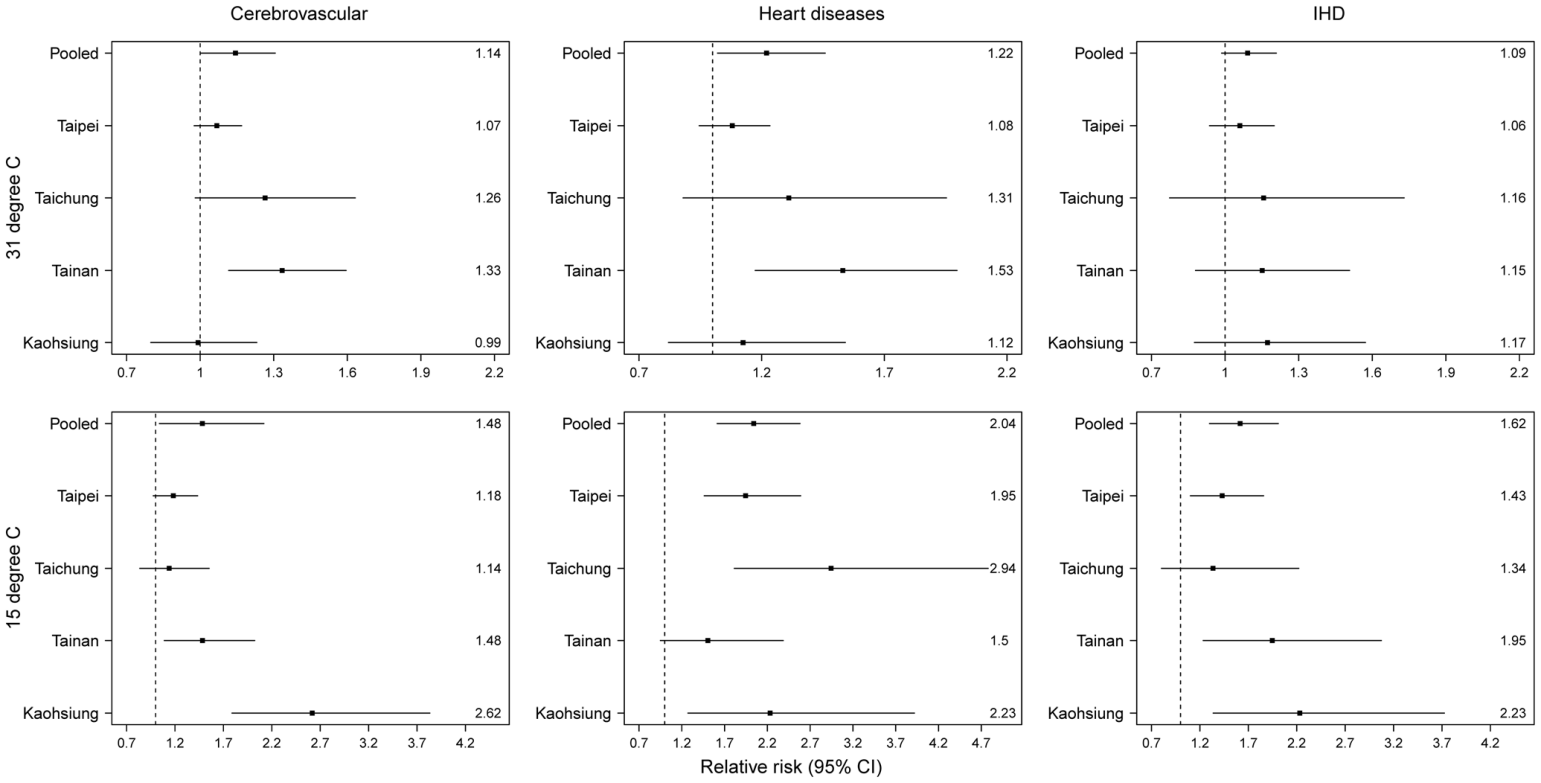
Metropolitan-specific and pooled relative risk estimates for the cumulative 21 days at 15°C and cumulative 4 days at 31°C compared to a centered temperature of 27°C using DLNM, controlling for the consecutive days of temperature extremes, daily city-specific averages of PM_10_, NOx, O_3_, and RH, daily mortality from pneumonia and influenza, holiday effects, day of a week, and long-term trends. Pooled risk estimates were obtained by random-effect meta-analysis.

Figure 6 summarizes the cumulative 4-day RRs of mortality associated with temperatures of 31°C (top row) and 21-day RRs of mortality associated with temperatures of 15°C (bottom row) using random-effect meta-analyses. At 31°C, the cumulative 4-day RRs of mortality comparing to that at the centered temperature was significant for cerebrovascular diseases (RR = 1.14; 95% CI: 1.00, 1.31) and heart diseases (RR = 1.22; 95% CI: 1.02, 1.46), but not for IHD (RR =  1.09; 95% CI: 0.99, 1.21). At 15°C, the cumulative 21-day RRs of mortality comparing to that at the centered temperature was significant for cerebrovascular diseases with an RR of 1.48 (95% CI: 1.04, 2.12), heart diseases with an RR of 2.04 (95% CI: 1.61, 2.59), and IHD with an RR of 1.62 (95% CI: 1.30, 2.01).

### Risks associated with prolonged extreme temperatures

Except mortality from IHD in Taipei, heart disease in Taichung, and cerebrovascular diseases in Kaohsiung, we did not identify any significant risks for extreme heat and cold events. As the population was exposed to the 97^th^ percentile extreme temperature for >8 day or longer, the RR for mortality from heart diseases was 1.68 (95% CI: 1.07, 2.63) in Taichung, and for mortality from cerebrovascular diseases was 1.33 (95% CI: 1.07, 1.66) in Kaohsiung. The RR for mortality from IHD was 1.27 (95% CI: 1.02, 1.58) when the population in Taipei was exposed to the 97^th^ percentile extreme temperature for 6–8 days.

### Risks associated with levels of air pollutants

This study did not identify any significant effect of PM_10_, NOx, and O_3_ on cause-specific CVD mortality after considering the prolonged effects (lag day 0-20) of extreme temperatures.

## Discussion

Current analyses revealed that low temperature caused greater adverse effects on mortality from cardiovascular diseases than that of high temperature in these four metropolitans in the subtropical area. Mortality risk is particularly higher for mortality from heart diseases (RR =  2.04; 95% CI: 1.61, 2.59) summarized by meta-analyses. In addition, risk of mortality from cardiovascular diseases generally peaked at lag 0 and lag 3 to 4 as population exposed to extreme heat (>31°C) and cold (<15°C), respectively. Moreover, risk of mortality from cardiovascular diseases was significantly associated with extreme heat event lasting for more than 5 days, but the risks varied with study areas and causes of mortality.

Excess mortality due to cardiovascular diseases was the main reason for deaths caused by high temperature [32]. Basu *et al.* reported that those dying from cardiovascular diseases, including IHD, congestive heart failure, and myocardial infarction (MI), were associated with increased temperature [5]. In addition, elevated mortality from cardiovascular diseases is associated with low temperature, which increases systolic and diastolic blood pressures, platelet count and serum low density [7]. Variations of temperature-related biological markers was associated with excess cardiovascular mortality in cold weather [33]. Bhaskaran *et al.* indicated that lower temperature could significantly increase short-term risk for incidence of MI [34]. American’s 50 cities study found mortality increases associated with both extreme heat and cold for MI and cardiac arrest deaths [35]. However, some other ecological studies reported null findings, in which Russian and European studies revealed that the mortality from cardiovascular diseases and IHD were not associated with low temperature [11], [36].

This study evaluated heat and cold effects on cause-specific CVD mortality, involving cerebrovascular diseases, heart diseases, and IHD. We investigated these cause-specific deaths based on their biological mechanisms known with temperature variations [12], [37]. Our findings were similar with some reports about Taiwanese population that mortality rates of CVD increased significantly after the cold surges [17]. Extreme low temperature rather than high temperature had greater impacts on the mortality from cardiovascular diseases, especially for the elderly, in Taiwan [18], [19]. Possible explanations from multicity studies that indicated cold effects were most significant in warmer regions [38], [39] or areas with moderate climates in winter [40]. On the other hand, in terms of heat effect, an American’s 43 communities study found that mortality impacts and effect modification by heat wave characteristics were more pronounced in the Northeast and Midwest regions compared with the South region [41]. Populations in subtropical areas are accustomed to hot and humid weather that would take adaptation as they exposed to high temperature.

IHD has become one of the leading causes of deaths around the world [12]. Significant inverse associations for cold effect were observed between IHD mortality and climate index in England [42], and ambient temperature in Japan and China [9], [43], which were similar with our findings. However, according to a study conducted in 15 European cities, they reported a 1°C decrease in temperature was associated with 1.72% and 1.25% increase in mortality from CVD and cerebrovascular disease [20]. Our findings revealed higher mortality risk than theirs. Although our study temperature indicator and composition of mortality from heart diseases were different, the association between them was approximately identical.

Investigators have reported heat and low temperature have different lag effect. Previous studies usually underestimated cold-related mortality risk, because they neglected to address prolonged effects [44], [45]. In general, lagged effect in cold days lasted longer than that in hot days [13]. Hot effects were generally acute and may last for three days, and followed by mortality displacement for cardiovascular deaths [46]. Xie *et al.* reported that the association between mortality and extreme cold events could last for up to 4 weeks for population in southeastern China [27]. This study adopted two different lag time courses, *i.e.* maximum 4 (lag day 0 to 3) and 21 days (lag day 0 to 20), to identify the corresponding delayed effect for extreme heat and cold on cause-specific CVD mortality. We identified mortality risks for cardiovascular diseases associated with extreme heat were generally highest at lag 0 to 1, but mortality risks associated with extreme cold were peaked at lag 3 to 4. This finding was similar with a U.S. study that reporting the effect of high temperature was restricted to the day of the death (lag 0) or the day before exposure (lag 1), but the effect of low temperature persisted for days [47]. An Austria study reported that heat effect was observed only for lag 0-1 and cold effect was for lag 10-15 [13]. This is the first study to analyze the relationship between cause-specific CVD mortality and temperature in subtropical areas using DLNM, properly evaluating the nonlinear association and cumulative risks related to ambient temperature at various lag days. The phenomenon for temperature lag effect is quite specific, and it indeed provided useful information for emergency public health response. However, underlying physiological, socioeconomical and environmental mechanisms for various lag effects for extreme heat and cold remain unclear to date, making further studies warranted.

Although the pooled mortality from cardiovascular diseases did not show significant association with prolonged extreme temperature events in the present study, elevated mortality from IHD in Taipei, heart disease in Taichung, and cerebrovascular diseases in Kaohsiung were specifically found significant for their associations with extreme heat events lasting for more than 5 days. The finding implied that risks of cardiovascular mortality varied with study area and cause of mortality. The estimated RRs were affected by latitude, demographics, social-economic and medical factors and showed city-specific associations [37], [48]. Meanwhile, this study did not identify any significant RR for cause-specific CVD mortality in association with first extreme heat and cold events in a year (detail not shown).

Exposure of air pollutants, including ozone, particulate matter, and NOx, also increases the mortality for vulnerable population. Evaluating the modifying role of ozone on temperature-related health impacts has been suggested crucial [49] and acute health effects of air pollution might vary by temperature level [50]. However, American’s 9 cities study provided evidence of increased mortality due to elevated apparent temperature exposure, with no confounding or effect modification due to air pollution [51]. This study had similar findings implying robust evidence for temperature effect on cardiovascular mortality.

This study has several limitations. First, temperature exposure was not measured at individual level. Ecological study design lacks information on personal exposure to the extreme high or low temperature events or air pollutions. Secondly, modifications from other socioeconomic factors, such as the usage of air conditioning or heater, marital status, and household income were not assessed because of the absence of individual information.

Climate change is leading to increasing frequency and intensity of extreme temperature events. Better understanding the patterns of CVD mortality associated with extreme temperatures is an important public health subject in dealing with health consequences resulting from global climate changes. There is an urgent need to conduct more large-scale, prospective, community-based and international studies to characterize the related risk factors in temperature-CVD mortality association [15].

## Conclusion

Extreme heat and cold significantly increased RRs of mortality from cardiovascular diseases. Low temperature was associated with greater risk of cause-specific mortality in comparison with that of high temperature in subtropical areas. Adverse effects of extreme temperature are acute and generally presented in the beginning of exposures. The risks of specific cardiovascular mortality varied with study areas and causes of mortality.
